# Predicting quality of life during and post detention in incarcerated juveniles

**DOI:** 10.1007/s11136-019-02160-6

**Published:** 2019-03-14

**Authors:** E. L. de Ruigh, A. Popma, J. W. R. Twisk, R. W. Wiers, H. S. van der Baan, R. R. J. M. Vermeiren, L. M. C. Jansen

**Affiliations:** 1 Department of Child and Adolescent Psychiatry, Amsterdam UMC, Vrije Universiteit Amsterdam, De Boelelaan 1117, Amsterdam, The Netherlands; 2 Institute of Criminal Law & Criminology, Faculty of Law, Leiden University, Leiden, The Netherlands; 3 Department of Epidemiology and Biostatistics, Amsterdam UMC, Vrije Universiteit Amsterdam, De Boelelaan 1117, Amsterdam, The Netherlands; 4 Department of Developmental Psychology, University of Amsterdam, Amsterdam, The Netherlands; 5 Department of Child and Adolescent Psychiatry, Curium-LUMC, Leiden University Medical Center, Leiden, The Netherlands

**Keywords:** Quality of life, General functioning, Juvenile offender, Trauma, Respiratory sinus arrhythmia

## Abstract

**Purpose:**

Besides reducing recidivism, juvenile justice institutions aim to rehabilitate juvenile delinquents, in order for them to reintegrate in society. As such, improving quality of life (QoL), especially post detention, is an important treatment goal. However, research is primarily focused on recidivism as an outcome measure for juvenile detention. The aim of the current study is therefore to describe and predict QoL of detained young offenders up to 1 year after an initial assessment, and to examine whether QoL differs between youth who are still detained versus released.

**Methods:**

A sample of 186 juveniles admitted to juvenile justice institutions in the Netherlands was assessed within the institution (initial assessment/T0), using psychosocial and neurobiological factors as predictors (self-control, treatment motivation, trauma, mental health problems, respiratory sinus arrhythmia). QoL (MANSA), as well as substance use (alcohol, cannabis) and daily activities (education, work) were assessed at first, second, and third follow-up (respectively 2.5 months, 4.5 months, and 12 months after T0).

**Results:**

QoL increased from first to third follow-up, and was higher for individuals who were no longer detained. The model that best predicted higher QoL upon follow-up consisted of lower trauma and stronger parasympathetic nervous system reactivity. The effects of the predictors did not differ between the various follow-ups, nor between individuals who were or were not detained.

**Conclusion:**

Methods incorporating trauma-sensitive focus and relaxation techniques in treatment protocols in juvenile justice institutions may be of added value in improving the general functioning of these individuals.

## Introduction

Delinquent behavior in detained youths is often accompanied by a variety of psychosocial and mental health problems [1]. Prisoners’ mental health is substantially worse in comparison to the general population [2]. Problems experienced by delinquent juveniles likely existed prior to admission and often persist post release [3–6]. Furthermore, incarceration itself has been shown to have a negative, iatrogenic impact [7, 8]. A review by Lambie and Randell [8] summarized that incarceration results in inferior education, as well as negative effects on social relationships, mental health, and physical health, thereby compromising reintegration. Cumulative risk behaviors after release further compromise the youngster’s future [9], impacting their well-being far into adulthood. Moreover, the transition back to the community is often concurrent with transitioning into adulthood. These juveniles are thus at risk for a range of negative outcomes [10].

The purpose of admission to a secure juvenile institution is a combination of punishment, providing increased safety for society, providing a deterrent for future offending, and rehabilitation. Of these, rehabilitation may be paramount, as incarceration alone does not reduce recidivism [11, 12]. To evaluate rehabilitative success, research has primarily focused on recidivism. The negative impact that offenders’ low general functioning has on society and the associated increased risk of recidivism render this factor equally important. General functioning can be subdivided into physical and mental health, daily life activities, education or employment, and quality of life (QoL) [13]. Diener and Suh [14] specify different indicators for QoL: economic, social, and subjective. The main focus of the current study is subjective QoL (further referred to as QoL). QoL reflects the person’s feelings about activities, life circumstances, and experiences. It is conceivable that when QoL is low, the need for support is greater. Indeed, many individuals require social or mental health services after release from a closed institution [6, 15]. The current study therefore aims to assess QoL in juvenile offenders during detention and post release.

Although often studied in mental health treatment and general psychiatry, QoL studies in offenders and forensic psychiatry are scarce [16–18]. Higher QoL has been associated with positive adjustment post release in adult offenders [16]. It is considered to be associated with a reduced risk of recidivism [19–21], while poor QoL is thought to increase this risk [16, 22, 23]. Less is known about QoL in detained juveniles. The few studies that were performed focused on youth in secure residential care, juvenile offenders’ health-related QoL, or QoL of detained girls exclusively [6, 24–26]. Girls with low QoL prior to detention were at an increased risk for mental health problems post release, increasing the risk of offending [25]. Only two studies scrutinized QoL in detained male juveniles. QoL scores were moderately high 1 year post release [6]. Furthermore, although QoL was higher for released individuals compared to those still admitted, release negatively affected satisfaction with family relations, social participation, and finances [27]. Therefore, release from a secure juvenile institution could be seen as a life event that might affect QoL [27].

It could be useful to be able to predict future QoL. Suitable candidate predictors likely promote or suppress beneficial post-release functioning. For example, lower mental health symptom scores have been associated with higher QoL [28]. Furthermore, youths persisting in antisocial behavior have been shown to exhibit deficits in self-control [29]. Self-control was found to be positively related to beneficial adjustment and negatively related to illegal substance misuse and recidivism post release [30]. Next, low treatment motivation has been related to recurrent problem behavior and violent recidivism [31]. Conversely, high treatment motivation could function as a buffer. Treatment motivation was found to be an important predictor of changes in QoL in somatic rehabilitation [32]. Finally, Belenko [33] outlines that former inmates have high rates of substance abuse post release, challenging successful integration. Several relevant environmental factors have also been identified. Negative life events in both juvenile delinquent and non-offender samples predicted psychosocial problems in young adulthood, and a decrease in psychosocial functioning over time [3, 34]. Longitudinal studies in adults show that life events can have a large impact on subjective well-being [e.g., 35]. Moreover, individuals who were witnesses as well as victims of violent crime showed poorest adjustment post release [36]. This applies to negative life events both prior to and during incarceration [37]. Next, employment was shown to improve both QoL and mental health [38]. During adolescence, working more than 20 h a week on average can reduce antisocial behavior, however, only in combination with regular school attendance [39]. Unfortunately, formerly incarcerated individuals face considerable barriers to educational and vocational attainment [40, 41], while low levels of academic performance are highly correlated with recidivism [42]. Failure to address post-release general functioning greatly reduces the likelihood that released inmates will be able to obtain and hold jobs, resulting in high odds of early relapse and recidivism [43]. Employment, mental health, and education—other aspects of general functioning—all show positive associations with QoL [28, 38, 39, 42]. It is therefore important to include the factors listed above when examining QoL in juvenile offenders.

Neurobiology is increasingly seen as an important factor in comprehending antisocial behavior [44–46]. Similarly, neurobiological factors may benefit the prediction of positive development. The parasympathetic branch (PNS) of the autonomic nervous system is a neurobiological system that is thought to be associated with emotion regulation abilities and prosocial behavior. Inhibitory effects of the PNS allow for a wide range of adaptive, prosocial behaviors [47]. A longitudinal study showed that children with greater PNS suppression were socially preferred by their peers [48]. This was mediated by better social skills for boys and girls, and fewer behavior problems for boys. Moreover, capacity for physiological regulation can function as a buffer against adverse consequences [community sample of children; 49]. Finally, there are indications that higher (baseline) PNS activity could protect against aggression when a low-quality environment is present [50]. This makes PNS activity a suitable candidate for predicting positive general functioning and QoL.

The first aim of the current study was to describe the development of QoL of detained juveniles up until 1 year after initial assessment (T0). It was examined whether differences in QoL exist between youth detained at follow-up and juveniles released from the institution. The second aim was to explore whether QoL at follow-up could be predicted based on relevant candidate predictors, measured at T0 inside the institution (self-control, treatment motivation, mental health problems, negative life events, PNS activity). It was expected that higher self-control, treatment motivation, and RSA (reactivity) would have a positive influence on QoL, and that a higher degree of mental health problems and trauma would negatively affect QoL. The possible influence of substance use, daily activities (employment, education), and detention status (detained/released) upon follow-up was assessed as well.

## Method

### Participants

Participants were recruited from five juvenile justice institutions in the Netherlands, where they were awaiting trial, served a juvenile detention sentence, and/or attended forensic psychiatric treatment. T0 data were collected during detention, in a collaboration between the department of Child and Adolescent Psychiatry of the Amsterdam UMC, Vrije Universiteit and the department of Developmental Psychology of the University of Amsterdam. Exclusion criteria were unwillingness or inability to participate or sign the informed consent, insufficient knowledge of the Dutch language, cardiac problems that could interfere with the measurement of heart rate or heart rate variability (e.g., arrhythmia or asthma), or an inability to understand the instructions and questionnaires as brought to our attention by institution staff. The participants were compensated for their time with a €5 incentive. Before testing, participants (and when < 18 also parents/caregivers) signed an informed consent document. This study was approved by the Ethics Committee of the University of Amsterdam and performed in accordance with the ethical standards described in the 1964 Declaration of Helsinki.

Out of 401 participants, a total of 186 male juveniles and young adults were included in the current study, ranging from 14.75 to 24.45 years (*M* = 18.58, SD = 1.63). Participants with missing data on ≥ 1 predictor variables (*n* = 162) were excluded from the analyses. The missing values stemmed from missing questionnaires, and therefore it was decided not to impute these values. Furthermore, girls (*n* = 8) and juveniles that did not participate in any of the follow-up measurements (*n* = 45) were excluded. Individuals who did not participate in any of the follow-up measurements were compared to participants that completed one or more follow-up measurements. They did not differ on age, socioeconomic status (SES), ethnicity, or level of education, nor did they differ on the predictors self-control, trauma, treatment motivation, or RSA (rest and reactivity). However, the subgroup that did participate in follow-ups (*M* = 7.27, SD = 4.52) reported more mental health problems (BPM-Y) than the subgroup that did not participate (*M* = 5.49, SD = 4.06; *t* (229) = − 2.43, *p* < 0.05).

Most participants in the final sample were of non-western descent (62.9%; defined as one or both parents being born in a non-western country, with the Dutch Antilles and Morocco being the most frequent). The remaining participants were of western descent (33.8% with the two most frequent countries/regions being the Netherlands and Western Europe). For 3.2%, no information on the place of birth of one or both parents was available. Regarding SES, 25.8% had a low SES, 65.6% an average SES, and 4.3% a high SES. SES could not be determined for 4.3%. The majority of participants (68.3%) completed vocational or higher secondary education, and 31.7% competed primary or lower secondary education. Participants stayed an average of 395 days (range 21–1898 days) in the institutions.

### Measures

An overview of the research instruments used and the timeline of their administration is presented in Table 1.

**Table 1:** Overview of measures at T0 and at the first, second and third follow-up assessments

T0	Follow-up assessments (T1–3)*
Adolescent Treatment Motivation Questionnaire	Daily activities (work, education)
Brief Problem Monitor-Youth	Manchester Short Assessment of Quality of Life
Brief Self-Control Scale	Substance use frequency
Childhood Trauma Questionnaire-short form	
Respiratory sinus arrhythmia (baseline & reactivity)	

*First follow-up (T1) 2.5 months, second follow-up (T2) 4.5 months, third follow-up (T3) 12 months after T0

The *Adolescent Treatment Motivation Questionnaire* (ATMQ) is an 11-item self-report instrument measuring treatment motivation. It was derived from the MTQ, based on the TTM of Prochaska and DiClemente [51]. Internal consistency reliability was good (Cronbach’s alpha 0.84) for a sample of adolescents in Dutch secure juvenile facilities [52].

The *Brief Problem Monitor-Youth* [BPM-Y; 53] measures potential problems along three dimensions, Internalizing, Externalizing, and Attentional Problems. The items are drawn from the Child Behavior Checklist for Ages 6–18 (CBCL/6-18), Teacher’s Report Form (TRF), and Youth Self-Report (YSR) [53]. The BPM-Y was shown to have adequate test–retest reliability and validity [54], and showed good psychometric properties in a Norwegian sample of children and adolescents [55]. Higher BPM-Y scores are indicative of more problems. The current study only used the youth self-report version of the BPM.

The *Brief Self-Control Scale* [BSCS; [56] was developed to assess dispositional self-control as it is conceptualized by contemporary theoretical perspectives [57]. The BSCS has shown good reliability and validity [30]. The participants provided answers based on the past 6 months.

The short form of the *Childhood Trauma Questionnaire* [CTQ-SF; 58] is a standardized, retrospective 28-item self-report inventory that measures the severity of childhood trauma (Emotional Abuse, Physical Abuse, Sexual Abuse, Emotional Neglect and Physical Neglect). The CTQ is validated for adolescent psychiatric patients [58] and male and female street youth [59]. The 28-item version is validated for multiple populations [60]. Item 24 was removed because experience and research showed a low validity of this item. Higher scores mean higher levels of trauma.

In order to map *daily activities*, participants were asked whether they had (paid or unpaid) work, what kind of work, and the amount of hours per week. The same was done for education, what kind of education, and the amount of hours per week. Only the dichotomous variables work (yes/no) and education (yes/no) were used for the analyses.

Heart rate variability (HRV), in the form of *respiratory sinus arrhythmia* (RSA), was measured using the VU-Ambulatory Monitoring System [VU-AMS; 61]. Data were analyzed with VU-AMS software and data analysis support was offered by the VU-AMS department of VU University. Placement of the electrodes was done according to the VU-AMS manual (http://www.vu-ams.nl/support/instruction-manual/). RSA reactivity was measured during two emotional film clips [‘The Champ,’ 62, 63, ‘Mohamed,’ 63], which were counterbalanced to assert that any differences could only be attributed to differences in the film clips presented. Preceding each film clip, participants viewed 1 min of an aquatic video while baseline functioning was measured (Coral Sea Dreaming, Small World Music Inc.). RSA values for both film clips and baselines were averaged, and then change scores were created subtracting baseline averages from selected target episodes of the film clips. This resulted in baseline as well as reactivity measures for PNS (/RSA) activity. The emotion evocation task and data preparations are described in more detail elsewhere [64]. Research has shown that amplitude of RSA—an index of PNS activity—is an accurate indicator of the efferent influence of the vagus on the heart [65].

The *Manchester Short Assessment of Quality of Life* [MANSA; 66] is a 13-item questionnaire that assesses QoL. It was derived from the Lancashire Quality of Life Profile [66]. The psychometric properties of the MANSA appear to be satisfactory [67]. A higher score represents better QoL. Due to an error in the questionnaire software, the question on satisfaction with the participants’ relationship was not included, resulting in 12 questions for the current study.

An indication of *substance use frequency* was obtained by means of self-reports of alcohol and cannabis use. The *Alcohol Use Disorder Identification Test* (AUDIT) was developed by the World Health Organization as a self-report screening instrument for hazardous and harmful alcohol use [68]. The AUDIT was shown to be reliable and valid [69] and includes items on alcohol consumption (3), dependence symptoms (3), and problems related to alcohol use (4) [70]. The *Cannabis Use Disorder Identification Test* (CUDIT) was developed as a 10-item instrument that would identify individuals using cannabis problematically [71]. A revised CUDIT-R was developed containing 8 items, and was shown to be a reliable and valid screening test [71]. The current study used the revised CUDIT-R. For both AUDIT and CUDIT-R, participants provided answers based on the past year; however, for the first two follow-up assessments, this was done for the past 2 weeks. Only the frequency of use of the substance was used for analyses.

### Procedure

Juveniles were eligible for participation after they had been in the institution for at least 3 weeks. During this time, the institution performed its own diagnostic research with juveniles, making further external research too demanding. The first test session (T0) took place under the supervision of a researcher in a test room within the institution. Participants were connected to the VU-AMS device, and electrodes (ECG Micropore electrodes H98SG) were placed according to the instructions in the VU-AMS manual (http://www.vu-ams.nl/support/instruction-manual/). For the next 10 min, participants filled in questionnaires on the computer to acclimatize to the equipment, while parameters of the autonomic nervous system (ANS) were measured as a natural baseline (acclimatization period). Participants were asked not to touch the electrodes and to sit as quietly as possible. ANS parameters were then measured while the participants performed tasks on a computer (DELL Latitude E5550 type laptop). These tasks consisted of another baseline measurement during a 5-min rest protocol [72], a countdown task [73], and viewing of two film fragments (Mohammed, The Champ), all interspersed with 1-min baselines. After completion of the ANS measurements, the participants were disconnected from the VU-AMS device and asked to collect saliva in a plastic tube. They continued with questionnaires and tasks on the computer for the remainder of the session. The total session lasted approximately 90 min. Afterwards participants were reminded of the follow-up assessments. If they had consented, they were asked to write down their contact details to enable contact once they had been released from the institution.

Three follow-up assessments were conducted at 2.5 months, 4.5 months, and 12 months after initial participation (i.e., T0). Mean days between T0 and follow-up were 91.6 days (SD = 18.92) for the first, 163 days (SD = 30.14) for the second, and 451.53 days (SD = 60.43) for the third follow-up. Participants were contacted by telephone and asked if they would like to take part in the follow-up interview. In case of consent, questionnaires were conducted. Questions were formulated in such a way that participants did not have to express sensitive information out loud. If participants (at that moment) were unable to participate in the follow-up measurement, they were asked if they objected to being contacted at a later time. The questionnaires that were administered are described above. Additionally, two questionnaires were administered concerning the participant’s friendships and self-reported recidivism. Because these questionnaires are not included in the current study, they are not described in further detail here. The duration of the follow-up assessments was 10 min for the first and second, and 15 min for the third follow-up. Participants were compensated for their time with a gift voucher of five euros, 10 euros, and 15 euros for the first, second, and third follow-up, respectively. Upon completion, participants were sent a link (via e-mail) where they could download the gift voucher.

### Statistical analyses

Data were analyzed using IBM SPSS statistics version 22. For the outcome measure QoL (MANSA), and possible confounders (substance use, daily activities, detention status), descriptives were calculated for all three follow-up measurements.

With linear mixed model analysis, first the change in QoL (MANSA) over time was analyzed. Possible differences in QoL between detained and released juveniles were examined with *t* tests. Subsequently, to determine the predictive value of the separate predictors for QoL, univariable mixed model analyses were performed with each predictor: treatment motivation (ATMQ), self-control (BSCS), trauma (CTQ-SF), mental health problems (BPM-Y), RSA in rest, and RSA reactivity. Then, multivariable mixed model analyses were performed to obtain the best predictive model of the predictors for QoL. A backwards selection strategy was employed, meaning that in separate multivariable mixed model analyses the predictors with the highest *p* value (*p* > 0.10) were removed from the model. Analyses were repeated with correction for control variables (daily activities: work, education; substance use: alcohol, cannabis; detention status: detained/released). Finally, interactions with time and detention status were explored for all predictors.

Mixed model analysis was used to take into account the dependency of the observations within the participants. Besides that, mixed model analysis is capable of dealing with missing data. This was important for the current analyses, as the dataset from this study contained a substantial number of missing data. Of the total 186 participants, 83.87% (*n* = 156) completed the first, 76.88% (*n* = 143) completed the second, and 55.91% (*n* = 104) completed the third and final follow-up measurement.

## Results

Descriptive statistics for QoL average scores, predictors, and control variables (daily activities: work, education; substance use: alcohol, cannabis) for all measurements are shown in Table 2. Mixed model analysis showed the increase in average QoL over time was significant from the first to the third follow-up (*p* < 0.001). At the first, second, and third follow-up measurements, 101 (54.3%), 78 (41.9%), and 33 (17.7%) participants in the respective follow-up assessment were still in detention. QoL differed significantly for juveniles who were detained versus released: participants in detention scored on average lower than released participants (see Table 3). Therefore, detention status was treated as additional control variable in univariable and multivariable mixed model analyses. Furthermore, for a subset of the current sample, we examined whether QoL at follow-up (T1–T3) differed between participants with a first offense (11.3%, *n* = 21) and multiple offenses (34.4%, *n* = 64; missing: *n* = 101); however, this was not the case (all *p* > 0.1).

**Table 2:** Descriptives for predictor variables at T0, average scores for QoL at follow-up, and whether participants reported work, education, or substance use upon follow-up assessments

	Mean	(SD)	Min	Max
Treatment motivation	26.24	(6.75)	15.00	43.00
Self-control	44.98	(7.79)	25.00	64.00
Mental health problems	7.27	(4.52)	0.00	22.00
Trauma	41.69	(11.51)	29.00	107.00
lg RSA rest	1.85	(0.23)	0.99	2.46
RSA reactivity	− 4.82	(20.89)	− 79.36	61.60
Quality of life first follow-up	5.41	(0.89)	1.50	7.00
Quality of life second follow-up	5.53	(0.84)	3.33	7.00
Quality of life third follow-up	5.72	(0.85)	3.25	7.00

	Follow-up 2.5 months		Follow-up 4.5 months		Follow-up 12 months	
	Job	Education	Job	Education	Job	Education
Yes^a^	36 (19.4%)	110 (59.1%)	36 (19.4%)	89 (47.8%)	39 (21.0%)	51 (27.4%)
No	119 (64.0%)	45 (24.2%)	106 (57.0%)	53 (28.5%)	65 (34.9%)	53 (28.5%)

	Alcohol	Cannabis	Alcohol	Cannabis	Alcohol	Cannabis
No	122 (65.6%)	91 (48.9%)	109 (58.6%)	81 (43.5%)	66 (35.5%)	44 (23.7%)
≤ 1 per month	21 (11.3%)	20 (10.8%)	25 (13.4%)	18 (9.7%)	24 (12.9%)	9 (4.8%)
2–4 per month	8 (4.3%)	8 (4.3%)	6 (3.2%)	8 (4.3%)	8 (4.3%)	9 (4.8%)
2–3 per week	5 (2.7%)	36 (19.4%)	3 (1.6%)	36 (19.4%)	5 (2.7%)	8 (4.3%)
≥ 4 per week	0 (0.0%)	0 (0.0%)	0 (0.0%)	0 (0.0%)	0 (0.0%)	30 (16.1%)

^a^Percentage of participants reporting a job or education at follow-up assessment

**Table 3:** Means scores, standard deviations, and *t* test statistics for differences in QoL between detained and released participants during follow-up

	Detained		Released		*t*	*p*
	*M*	SD	*M*	SD		
Quality of life follow-up 2.5 months	5.20	0.89	5.80	0.73	− 4.27	< 0.001
Quality of life follow-up 4.5 months	5.25	0.73	5.87	0.83	− 4.75	< 0.001
Quality of life follow-up 12 months	5.41	0.81	5.86	0.83	− 2.59	< 0.05

### The predictive value of individual predictors

Because of the exploratory nature of the analyses, a cut-off value of 0.10 (*p* value) was used with respect to significance level. Univariable mixed model analyses for the separate predictors showed that self-control, trauma, and mental health problems significantly predicted QoL (see Table 4). These results remained after controlling for the control variables (see Table 5).

**Table 4:** Results from univariable and multivariable analyses

	Univariable analyses			Multivariable analyses		
	*β*	95% CI	*p*	*β*	95% CI	*p*
Treatment motivation	0.01	− 0.01 to 0.03	0.183			
Self-control	0.01	0.00 to 0.03	< 0.01			
Trauma	− 0.02	− 0.03 to − 0.02	< 0.001	− 0.03	− 0.03 to − 0.02	< 0.001
Mental health problems	− 0.03	− 0.06 to − 0.01	< 0.01			
RSA rest	0.11	− 0.26 to 0.48	0.555			
RSA reactivity	− 0.02^a^	− 0.06^a^ to 0.02^a^	0.349	− 0.04^a^	− 0.09 to 0.00^a^	< 0.10

*CI* confidence Interval, *RSA* respiratory sinus arrhythmia

^a^Due to the wide range of this variable, the value is multiplied by 10 to facilitate interpretation

**Table 5:** Results from univariable and multivariable analyses corrected for control variables (daily activities: work, education; substance use: alcohol, cannabis; detention status)

	Univariable analyses			Multivariable analyses		
	*β*	95% CI	*p*	*β*	95% CI	*p*
Treatment motivation	0.00	− 0.01 to 0.02	0.679			
Self-control	0.01	0.00 to 0.02	< 0.01			
Trauma	− 0.02	− 0.03 to − 0.01	< 0.001	− 0.02	− 0.03 to − 0.02	< 0.001
Mental health problems	− 0.03	− 0.05 to − 0.02	< 0.001			
RSA rest	0.13	− 0.20 to 0.45	0.438			
RSA reactivity	− 0.02^a^	− 0.05^a^ to 0.02^a^	0.312	− 0.05^a^	− 0.09^a^ to 0.01^a^	< 0.05

*CI* confidence Interval, *RSA* respiratory sinus arrhythmia

^a^Due to the wide range of this variable, the value is multiplied by 10 to facilitate interpretation

### The best predictive model for quality of life

Multivariable mixed model analyses with a backwards selection strategy resulted in a model with trauma and RSA reactivity as significant predictors for QoL (Table 4). The analysis was repeated for the final model with correction for daily activities (work, education), substance use (alcohol, cannabis), and detention status (detained/not detained). These results remained after controlling for the control variables (see Table 5). The explained variance was 12.17% for the model without, and 22.24% for the model with control variables.

### Interactions

As a final step, univariable interactions with time and detention status were explored for all predictors. No significant interactions were found between the predictors and time (*p* values for all interactions were > 0.28) or detention status (*p* values for all interactions were > 0.43).

## Discussion

The aim of this study was to describe quality of life (QoL) of detained juveniles, and to identify predictors of QoL, up until 1 year after initial assessment (T0). There was a slight but significant increase in QoL from the first to the third and final follow-up. Furthermore, QoL was higher for individuals who were no longer detained. When assessed separately, higher self-control, lower trauma, and less mental health problems at T0 were predictive of better QoL at follow-up, while RSA (rest and reactivity) and treatment motivation were not predictive of QoL. However, the multivariable model that best predicted QoL at follow-up consisted of the predictors trauma and RSA reactivity: lower trauma and stronger RSA reactivity predicted higher QoL. The effects remained, and were in fact marginally strengthened, when controlling for daily activities, substance use, and whether juveniles were detained or not. There were no interactions with time or detention status, meaning that the predictive value of the variables identified was stable over time and not informed by whether juveniles were detained or not during follow-up.

### Increased QoL post release

QoL was rated higher by individuals who were no longer in a juvenile justice institution. This is in accordance with the findings of an earlier study in a mixed sample of juveniles from youth residential care and juvenile justice institutions. Discharged juveniles reported greater satisfaction on most domains of QoL compared to juveniles who were still admitted [27]. Also in line with two previous studies [6, 27] is the finding that the observed QoL scores were moderately high. In the current study, there was a significant increase in average scores between the first and third follow-up. Since this increase was quite small, it is doubtful that this is clinically relevant. Average scores remained between the ‘mostly satisfied’ and ‘pleased’ with life outcomes. However, it is particularly noteworthy that the minimum scores clearly increased: after 4 months no scores under 3 (mostly dissatisfied) were given. It may be possible to achieve a larger increase in QoL by explicitly focusing on an individuals’ QoL goals (e.g., through QoL promotion) during treatment in a closed institution. Achieving a higher QoL is particularly important in view of the association between poor QoL and increased chances of delinquent behavior [16, 22, 23]. Notably, motivating youth to actively participate in treatment is often considered a challenge. Explicitly applying QoL promotion might enhance cooperation with treatment. QoL promotion aims to help individuals reduce the discrepancy between actual and desired QoL through treatment [74]. There are indications that QoL promotion during (community-based) treatment is associated with higher treatment completion [e.g., 75].

### Predicting QoL

It was expected that higher self-control, treatment motivation and RSA, and a lower degree of mental health problems and trauma would have a positive influence on QoL. The current results corroborate the directions of these associations. However, RSA in rest and treatment motivation were not predictive of QoL, and self-control and mental health problems were only predictive of QoL when assessed separate from the other factors. When the joint impact of the candidate predictors was examined, the best predictive model showed that only trauma and RSA reactivity significantly predicted QoL. It appears important to investigate factors in conjunction rather than examining isolated connections. This is particularly recommended within neurobiological research, where the interaction of different factors seems to be especially important in the development of behavior [e.g., 76].

These results show that experiencing childhood trauma (< 18 years) has a lasting impact on later QoL. Given the high numbers of reported childhood trauma before and during stay in an institution [e.g., 37], this seems to be an important factor to address in treatment, especially in view of the long-term impact that maltreatment has on psychosocial functioning [e.g., 3]. Treatment focused on childhood traumatic experiences is also important because of the impact that the behavior of these juveniles has on society. Maltreated youth placed out of their homes came into contact with the judicial system more than twice as often compared to maltreated youth who were not removed from their families [77]. Moreover, it appears that persistently neglected adolescents are significantly more likely to continue to reoffend compared to individuals without an official history of neglect [78]. Although the existence of trauma in a detained youth’s past can pose a challenge for treatment, trauma-sensitive work is of the utmost importance for this population [79].

A higher RSA reactivity score predicted better QoL. This would mean that juveniles with a diminished parasympathetic nervous system (PNS) response are at risk of lower QoL. There are indications in community samples and high-risk youth that RSA (reactivity) can be influenced through intervention [e.g., yoga and mindfulness; 80–83]. A review and meta-analysis in prisoners showed that participation in meditation or yoga is related to increased psychological well-being and improved behavioral functioning [84]. Mindfulness and yoga techniques were shown to lead to stress resilience, decreased perceived stress, and increased self-regulation and control in detained adolescents [85, 86]. The use of such techniques could therefore be a good addition to the treatment offered to youth in a closed setting.

### Study limitations

To our knowledge, this is the first study predicting QoL of juveniles admitted to a closed institution from a biopsychosocial perspective by combining psychosocial factors and neurobiological factors. A number of limitations should be considered when assessing the results of this study. First, due to missing values on questionnaires measuring the predictors at T0, the study sample was reduced to 186 participants. This was due to some of the instruments being included later on during the course of the study. Although the final sample was still relatively large, which can be challenging in samples of detained juveniles, follow-up research with other or larger samples is advisable to examine generalizability. Second, the timing at which juveniles participated on T0 within their detention period differed. Because the context of juveniles at the moment of admission to an institution is very heterogeneous, for example with regard to previous interventions or being previously detained, emphasis was placed on carrying out T0 during detention, rather than at the moment of (often stressful) admission. Third, not all participants took part in one or more follow-up measurements. With regard to attrition at follow-up, the retention rate (55% retention rate after 12-month follow-up) is still quite positive. Attrition is somewhat inherent in the target group; it is often difficult to contact juveniles once released from an institution. In order to limit the possible influence this has on results, a method of analysis was elected that is well capable of dealing with missings (see Method section). Fourth, all questionnaires in the current study were self-report questionnaires. There could be a possible risk of socially desirable answers, or there may be a potential for recall bias. In order to overcome this, future research could make use of, or self-report could be complemented by, the use of other report for potential predictors of QoL. Moreover, the measure for QoL was not administered at T0, and therefore baseline QoL was not controlled for in conducting the predictive analyses. Finally, our current model best able to predict QoL should not be considered as a true model of reality. Among the information collected in the current study, significant predictors for QoL were found. However, adding other information may alter which factors form the best predictive model for QoL.

### Implications and future recommendations

Juveniles with low satisfaction in certain areas of life could be at risk of persisting in their delinquent behavior. The current results can provide support to clinicians in setting adequate treatment goals. Future research must show whether the results can be generalized to other samples of detained or admitted juveniles and young adults. In the meantime, it could be of added value to use QoL as a tool in the treatment of juveniles admitted to a closed institution, in order to achieve an increase in QoL. This could be brought about immediately upon admission to an institution by establishing focus areas with regard to juveniles’ desired QoL. By monitoring and making interim adjustments to these factors during treatment, it may be possible to increase treatment motivation as well as QoL. With regard to the QoL predictors identified in this study, trauma and RSA reactivity, it is possible to apply interventions that are focused on these factors. While complete prevention of traumatic events (during detention) would be ideal, at least a clear focus on trauma in treatment is important in view of the connections with later poor psychosocial functioning and delinquent behavior. Finally, PNS (RSA) activity is susceptible to influence by intervention (e.g., mindfulness, yoga), leading to potential improvements in resilience.

## References

[1] Colins O (2009). Psychiatric disorders in property, violent, and versatile offending detained male adolescents. American Journal of Orthopsychiatry.

[2] Fazel S, Danesh J (2002). Serious mental disorder in 23 000 prisoners: A systematic review of 62 surveys. The Lancet.

[3] Basto-Pereira M (2016). Growing up with adversity: From juvenile justice involvement to criminal persistence and psychosocial problems in young adulthood. Child Abuse & Neglect.

[4] van der Molen E (2015). Girls’ childhood trajectories of disruptive behavior predict adjustment problems in early adolescence. Journal of Child Psychology and Psychiatry.

[5] van der Molen E (2013). Detained adolescent females’ multiple mental health and adjustment problem outcomes in young adulthood. Journal of Child Psychology and Psychiatry.

[6] Harder AT, Knorth EJ, Kalverboer ME (2011). Transition secured? A follow-up study of adolescents who have left secure residential care. Children and Youth Services Review.

[7] Gatti U, Tremblay RE, Vitaro F (2009). Iatrogenic effect of juvenile justice. Journal of Child Psychology and Psychiatry.

[8] Lambie I, Randell I (2013). The impact of incarceration on juvenile offenders. Clinical Psychology Review.

[9] Jahnukainen M (2007). High-risk youth transitions to adulthood: A longitudinal view of youth leaving the residential education in Finland. Children and Youth Services Review.

[10] Altschuler DM, Brash R (2004). Adolescent and teenage offenders confronting the challenges and opportunities of reentry. Youth Violence and Juvenile Justice.

[11] Andrews DA (1990). Does correctional treatment work? A clinically relevant and psychologically informed meta-analysis. Criminology.

[12] McGuire J (2002). Criminal sanctions versus psychologically-based interventions with offenders: A comparative empirical analysis. Psychology, Crime and Law.

[13] Baune BT (2010). The role of cognitive impairment in general functioning in major depression. Psychiatry Research.

[14] Diener E, Suh E (1997). Measuring quality of life: Economic, social, and subjective indicators. Social Indicators Research.

[15] Lovell D, Gagliardi GJ, Peterson PD (2002). Recidivism and use of services among persons with mental illness after release from prison. Psychiatric Services.

[16] Bouman YH, Schene AH, de Ruiter C (2009). Subjective well-being and recidivism in forensic psychiatric outpatients. International Journal of Forensic Mental Health.

[17] Ogloff JR, Davis MR (2004). Advances in offender assessment and rehabilitation: Contributions of the risk-needs-responsivity approach. Psychology, Crime & Law.

[18] van Nieuwenhuizen C, Schene A, Koeter M (2002). Quality of life in forensic psychiatry: An unreclaimed territory?. International Review of Psychiatry.

[19] Fisher D, Morgan J, Leeson S (2010). Working with juveniles with sexually abusive behaviour in the UK: The G-map approach. Current perspectives and applications in neurobiology: Working with young persons who are victims and perpetrators of sexual abuse.

[20] Psychology (2014). The Good Lives Model: New directions for preventative practice with children?. Crime & Law.

[21] Wylie LA, Griffin HL (2013). G-map’s application of the Good Lives Model to adolescent males who sexually harm: A case study. Journal of Sexual Aggression.

[22] Willis GM, Grace RC (2008). The quality of community reintegration planning for child molesters: Effects on sexual recidivism. Sexual Abuse.

[23] Willis GM, Ward T (2011). Striving for a good life: The good lives model applied to released child molesters. Journal of Sexual Aggression.

[24] Forrest CB (2000). The health profile of incarcerated male youths. Pediatrics.

[25] Van Damme L (2016). Quality of life in relation to future mental health problems and offending: Testing the good lives model among detained girls. Law and Human Behavior.

[26] Van Damme L (2015). Girls’ quality of life prior to detention in relation to psychiatric disorders, trauma exposure and socioeconomic status. Quality of Life Research.

[27] Barendregt C (2015). Stability and change in subjective quality of life of adolescents in secure residential care. The Journal of Forensic Psychiatry & Psychology.

[28] Priebe S (2010). Factors influencing subjective quality of life in patients with schizophrenia and other mental disorders: a pooled analysis. Schizophrenia Research.

[29] Monahan KC (2009). Trajectories of antisocial behavior and psychosocial maturity from adolescence to young adulthood. Developmental Psychology.

[30] Malouf ET (2014). The brief self-control scale predicts jail inmates’ recidivism, substance dependence, and post-release adjustment. Personality and Social Psychology Bulletin.

[31] Mulder E (2011). Risk factors for overall recidivism and severity of recidivism in serious juvenile offenders. International Journal of Offender Therapy and Comparative Criminology.

[32] Grahn B, Ekdahl C, Borgquist L (2000). Motivation as a predictor of changes in quality of life and working ability in multidisciplinary rehabilitation. Disability and Rehabilitation.

[33] Belenko S (2006). Assessing released inmates for substance-abuse-related service needs. Crime & Delinquency.

[34] Pagano ME (2004). Stressful life events as predictors of functioning: Findings from the Collaborative Longitudinal Personality Disorders Study. Acta Psychiatrica Scandinavica.

[35] Luhmann M (2012). Subjective well-being and adaptation to life events: A meta-analysis. Journal of Personality and Social Psychology.

[36] Boxer P, Middlemass K, Delorenzo T (2009). Exposure to violent crime during incarceration: Effects on psychological adjustment following release. Criminal Justice and Behavior.

[37] Dierkhising CB, Lane A, Natsuaki MN (2014). Victims behind bars: A preliminary study of abuse during juvenile incarceration and post-release social and emotional functioning. Psychology, Public Policy, and Law.

[38] Sneed Z (2006). Employment and psychosocial outcomes for offenders with mental illness. International Journal of Psychosocial Rehabilitation.

[39] Monahan KC, Steinberg L, Cauffman E (2013). Age differences in the impact of employment on antisocial behavior. Child Development.

[40] Abrams LS, Snyder SM (2010). Youth offender reentry: Models for intervention and directions for future inquiry. Children and Youth Services Review.

[41] Cavendish W (2014). Academic attainment during commitment and postrelease education-related outcomes of juvenile justice-involved youth with and without disabilities. Journal of Emotional and Behavioral Disorders.

[42] Katsiyannis A (2008). Juvenile delinquency and recidivism: The impact of academic achievement. Reading & Writing Quarterly.

[43] Taxman FS, Byrne JM, Young DW (2002). Targeting for reentry: Matching needs and services to maximize public safety.

[44] Popma A (2006). Hypothalamus pituitary adrenal axis and autonomic activity during stress in delinquent male adolescents and controls. Psychoneuroendocrinology.

[45] Beauchaine T (2008). Ten good reasons to consider biological processes in prevention and intervention research. Development and Psychopathology.

[46] Stadler, C., Poustka, F., & Sterzer, P. The heterogeneity of disruptive behavior disorders–implications for neurobiological research and treatment. *Frontiers in Psychiatry*, 2010. 1. 10.3389/fpsyt.2010.00021 PMC3059624 21423432

[47] Beauchaine TP, Gatzke-Kopp L, Mead HK (2007). Polyvagal theory and developmental psychopathology: Emotion dysregulation and conduct problems from preschool to adolescence. Biological Psychology.

[48] Graziano PA, Keane SP, Calkins SD (2007). Cardiac vagal regulation and early peer status. Child Development.

[49] Blandon AY (2008). Individual differences in trajectories of emotion regulation processes: The effects of maternal depressive symptomatology and children’s physiological regulation. Developmental psychology.

[50] Eisenberg N (2012). Differential susceptibility and the early development of aggression: Interactive effects of respiratory sinus arrhythmia and environmental quality. Developmental Psychology.

[51] van Binsbergen, M. (2003). Motivatie voor behandeling: Ontwikkeling van behandelmotivatie in een justitiële instelling: Garant.

[52] Van der Helm G (2013). Measuring treatment motivation in secure juvenile facilities. International Journal of Offender Therapy and Comparative Criminology.

[53] Achenbach T, Rescorla L (2001). Manual for the ASEBA school-age forms & profiles.

[54] Achenbach T (2011). Manual for the ASEBA brief problem monitor (BPM).

[55] Richter J (2015). Preliminary evidence for good psychometric properties of the Norwegian version of the Brief Problems Monitor (BPM). Nordic Journal of Psychiatry.

[56] Tangney JP, Baumeister RF, Boone AL (2004). High self-control predicts good adjustment, less pathology, better grades, and interpersonal success. Journal of Personality.

[57] Maloney PW, Grawitch MJ, Barber LK (2012). The multi-factor structure of the Brief Self-Control Scale: Discriminant validity of restraint and impulsivity. Journal of Research in Personality.

[58] Bernstein DP (1997). Validity of the Childhood Trauma Questionnaire in an adolescent psychiatric population. Journal of the American Academy of Child & Adolescent Psychiatry.

[59] Forde DR (2012). Factor structure and reliability of the childhood trauma questionnaire and prevalence estimates of trauma for male and female street youth. Journal of Interpersonal Violence.

[60] Bernstein, D. P., et al. (2003). Development and validation of a brief screening version of the Childhood Trauma Questionnaire. *Child Abuse & Neglect*. 27(2), 169–190. 10.1016/s0145-2134(02)00541-0 12615092

[61] de Geus E, Canli T (2014). Genetics of autonomic nervous system activity. The Oxford handbook of molecular psychology.

[62] Lovell D, Zeffirelli F (1979). The champ [Motion picture].

[63] De Wied M (2012). Verbal, facial and autonomic responses to empathy-eliciting film clips by disruptive male adolescents with high versus low callous-unemotional traits. Journal of Abnormal Child Psychology.

[64] de Ruigh, E., et al. Psychopathic traits and empathic functioning in detained juveniles: Withdrawal response to empathic sadness. *International Journal of Forensic Mental Health*.

[65] Porges S (2011). The Norton series on interpersonal neurobiology. The polyvagal theory: Neurophysiological foundations of emotions, attachment, communication, and self-regulation.

[66] Oliver J (1997). Measuring the quality of life of severely mentally ill people using the Lancashire Quality of Life Profile. Social Psychiatry and Psychiatric Epidemiology.

[67] Priebe S (1999). Application and results of the Manchester Short Assessment of Quality of Life (MANSA). International Journal of Social Psychiatry.

[68] Saunders JB (1993). Development of the alcohol use disorders identification test (AUDIT): WHO collaborative project on early detection of persons with harmful alcohol consumption-II. Addiction.

[69] Allen JP (1997). A review of research on the Alcohol Use Disorders Identification Test (AUDIT). Alcoholism.

[70] Boschloo L (2010). The performance of the Alcohol Use Disorder Identification Test (AUDIT) in detecting alcohol abuse and dependence in a population of depressed or anxious persons. Journal of Affective Disorders.

[71] Adamson SJ (2010). An improved brief measure of cannabis misuse: The Cannabis Use Disorders Identification Test-Revised (CUDIT-R). Drug and Alcohol Dependence.

[72] Scarpa A, Haden SC, Tanaka A (2010). Being hot-tempered: Autonomic, emotional, and behavioral distinctions between childhood reactive and proactive aggression. Biological Psychology.

[73] Wang P (2012). Psychopathic traits and physiological responses to aversive stimuli in children aged 9–11years. Journal of Abnormal Child Psychology.

[74] Williams D, Strean WB (2002). The transtheoretical model and quality of life promotion: Towards successful offender rehabilitation. Probation Journal.

[75] Williams D (1999). Quality of life recognition and promotion in offender rehabilitation. Corrections Compendium.

[76] Raine A (2002). Biosocial studies of antisocial and violent behavior in children and adults: A review. Journal of Abnormal Child Psychology.

[77] Ryan JP, Testa MF (2005). Child maltreatment and juvenile delinquency: Investigating the role of placement and placement instability. Children and Youth Services Review.

[78] Ryan JP, Williams AB, Courtney ME (2013). Adolescent neglect, juvenile delinquency and the risk of recidivism. Journal of Youth and Adolescence.

[79] Ford JD (2012). Complex trauma and aggression in secure juvenile justice settings. Criminal Justice and Behavior.

[80] Tyagi A, Cohen M (2016). Yoga and heart rate variability: A comprehensive review of the literature. International Journal of Yoga.

[81] Ditto B, Eclache M, Goldman N (2006). Short-term autonomic and cardiovascular effects of mindfulness body scan meditation. Annals of Behavioral Medicine.

[82] Fishbein D (2016). Behavioral and psychophysiological effects of a yoga intervention on high-risk adolescents: A randomized control trial. Journal of Child and Family Studies.

[83] Goldstein MR (2016). Improvements in well-being and vagal tone following a yogic breathing-based life skills workshop in young adults: Two open-trial pilot studies. International Journal of Yoga.

[84] Auty KM, Cope A, Liebling A (2017). A systematic review and meta-analysis of yoga and mindfulness meditation in prison: Effects on psychological well-being and behavioural functioning. International Journal of Offender Therapy and Comparative Criminology.

[85] Ramadoss R, Bose B (2010). Transformative life skills: Pilot study of a yoga model for reduced stress and improving self-control in vulnerable youth. International Journal of Yoga Therapy.

[86] Himelstein S (2012). Mindfulness training for self-regulation and stress with incarcerated youth: A pilot study. Probation Journal.

